# Engineered Resistance to Tobamoviruses

**DOI:** 10.3390/v16071007

**Published:** 2024-06-22

**Authors:** John Peter Carr

**Affiliations:** Department of Plant Sciences, University of Cambridge, Downing Street, Cambridge CB2 3EA, UK; jpc1005@cam.ac.uk

**Keywords:** mosaic virus, novel viruses, pathogen-derived resistance, RNAi, artificial microRNA, plant gene editing, NB-LRR protein, nanoparticle RNA delivery, plant disease, crop protection

## Abstract

Tobacco mosaic virus (TMV) was the first virus to be studied in detail and, for many years, TMV and other tobamoviruses, particularly tomato mosaic virus (ToMV) and tobamoviruses infecting pepper (*Capsicum* spp.), were serious crop pathogens. By the end of the twentieth and for the first decade of the twenty-first century, tobamoviruses were under some degree of control due to introgression of resistance genes into commercial tomato and pepper lines. However, tobamoviruses remained important models for molecular biology, biotechnology and bio-nanotechnology. Recently, tobamoviruses have again become serious crop pathogens due to the advent of tomato brown rugose fruit virus, which overcomes tomato resistance against TMV and ToMV, and the slow but apparently inexorable worldwide spread of cucumber green mottle mosaic virus, which threatens all cucurbit crops. This review discusses a range of mainly molecular biology-based approaches for protecting crops against tobamoviruses. These include cross-protection (using mild tobamovirus strains to ‘immunize’ plants against severe strains), expressing viral gene products in transgenic plants to inhibit the viral infection cycle, inducing RNA silencing against tobamoviruses by expressing virus-derived RNA sequences in planta or by direct application of double-stranded RNA molecules to non-engineered plants, gene editing of host susceptibility factors, and the transfer and optimization of natural resistance genes.

## 1. Prologue: How Tobamoviruses Went from a Threat to Crop Production to a Footnote in the History of Virology

**Figure 1:**
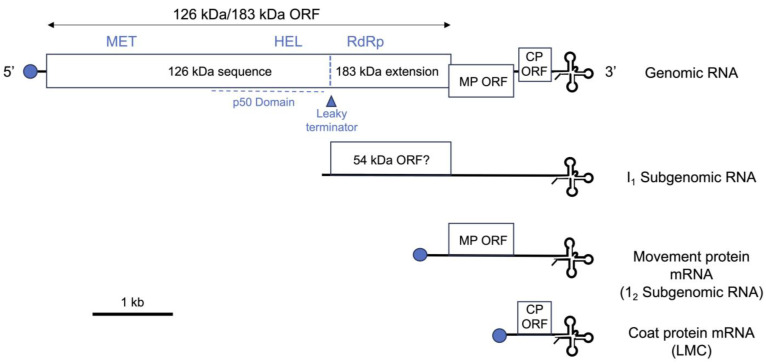
A diagram showing the organization of a representative tobamovirus (tobacco mosaic virus strain U1) genome indicating the viral genes and gene products mentioned in the text. Horizontal lines represent RNA and boxes represent open reading frames (ORFs) for known or putative viral proteins. The full length 183 kDa replicase protein is synthesized by read-through of a leaky termination codon at the end of the ORF encoding the 126 kDa protein, indicated with a triangle. The 126 kDa protein is a replicase component with methyltransferase (MT) and RNA helicase (HEL) domains, as well as an RNA silencing suppressor. The HEL domain lies within the ‘p50’ domain, which is the elicitor for the *N* gene-mediated hypersensitive response (refer to Figure 2). Both the 126 and 183 kDa proteins are synthesized by translation of the genomic RNA. The readthrough region of the 183 kDa protein contains the viral RNA-dependent RNA polymerase (RdRp) active site. The movement protein (MP) and coat protein (CP) gene sequences are synthesized by translation of subgenomic mRNAs, which are generated during infection. An additional subgenomic RNA (‘intermediate 1’: I_1_) encoding a putative 54 kDa protein is produced during infection. The putative 54 kDa protein would have the same sequence as the readthrough region of the 183 kDa protein, including its RdRp domain. The I_1_ RNA is translatable in vitro but it is not known if it has mRNA activity in planta. The subgenomic RNAs and the genomic RNA share identical 3′-teminal tRNA-like structures. The 5′ termini of the genomic, MP and CP mRNAs are capped (blue circles), and this may also be true for the I_1_ RNA. Not shown is a short ORF for a 4 kDa protein overlying the MP and CP ORFs, which is present in some tobamoviruses, and which is thought to be translated by means of a putative internal ribosome entry site.

The final two decades of the 19th century saw the discovery of tobacco mosaic virus (TMV) and the establishment of the ‘virus’ concept, i.e., infectious agents distinct from other microbes, and the establishment of virology as an important new field of research [1,2]. The initial impetus to the discovery of TMV arose from the need to understand and combat the mosaic disease of tobacco (*Nicotiana tabacum* L.), which was causing significant material and financial losses [2,3]. TMV and the many other tobamoviruses subsequently discovered, including, for example, tomato mosaic virus (ToMV) and pepper mild mottle virus (PMMoV), continued for many years to cause serious crop losses, especially for tomato (*Solanum lycopersicum* L.) and pepper (*Capsicum annuum* and related species), respectively [4,5,6]. Tobamoviruses are hard to control because they are highly infectious and spread easily by mechanical transmission, also called contact transmission, through the abrasion and wounding of susceptible plant tissue in the presence of inoculum. Their rod-shaped particles (virions) provide protection for their single-stranded positive-sense RNA genomes (Figure 1) and are extremely stable. The stability of tobamovirus virions makes disinfection of agricultural tools and growth facilities difficult, and it enables infectious tobamovirus inoculum to persist in both liquid and frozen water, in soil, within dead plant material and tobacco products, as well as on the clothing of agricultural workers [4,7,8].

In addition to the agricultural importance of tobamoviruses, academic studies of these viruses provided important insights into viral particle structure, macromolecular assembly and genome organization, viral and plant gene expression, plant–pathogen interactions, and viral evolution, among many other aspects of plant, viral and molecular biology [1,9,10]. During the first years of the twenty-first century, TMV emerged as an important tool for the co-option of its virions as vehicles for drug delivery and as structural components in bio-based nanotechnology [11]. More surprising recent applications for tobamoviruses include the use of PMMoV as a tracer for sewage contamination in water supplies [12].

However, by the turn of the twenty-first century and well into its second decade, tobamoviruses had become less important threats to commercial tomato cultivation in developed countries. Indeed, tobamoviruses were barely mentioned or not at all mentioned in some authoritative reviews on tomato viral diseases published during this period (for example, see refs. [13,14]). Instead, cucumber mosaic virus (CMV) and its satellite RNAs, tomato spotted wilt virus, or emerging viral pathogens, including pepino mosaic virus (PepMV), tomato yellow leaf curl virus, and viruses belonging to the *Torradovirus* and *Crinivirus* genera, were by that time considered to be greater threats to tomato production than tobamoviruses [13,14,15,16].

The decline in the importance of tobamoviruses as threats to tomato cultivation occurred in two stages. The first was the development of cross-protection, an approach using intentional inoculation or ‘immunization’ of seedlings of susceptible varieties with mild, TMV or ToMV mutant strains to inhibit subsequent infection by more damaging wild-type virus strains [4,17]. Subsequently, the use of cross-protection diminished as commercial tomato lines were improved by introgression of the resistance genes *Tm-1* from *S. habrochaites* (syn. *Lycopersicon hirsutum*), or the allelic *Tm-2* and *Tm-2^2^* genes from *S. peruvianum* (syn. *L. peruvianum*) [18]. *Tm-1* and *tm-1* encode similar 754 amino acid proteins, which differ at 25 residues. The Tm-1 protein but not the tm-1 protein binds to the RNA helicase domain of the 126/183 kDa protein (Figure 1) of ToMV and TMV and inhibits replicase complex formation. In contrast, the tm-1 protein has no effect on ToMV or TMV replication, but both the tm-1 and Tm-1 factors prevent multiplication in tomato of the tobamoviruses tobacco mild green mosaic virus (TMGMV) and PMMoV. Thus, *Tm-1* and *tm-1* help to delineate tobamovirus host ranges, and the contrasting interactions of their protein products with replicase proteins explain why TMGMV and PMMoV cannot infect tomato [19,20]. Unfortunately, TMV and ToMV mutants that can evade resistance conferred by *Tm-1* arise frequently, which limits the effectiveness of this resistance gene. However, this problem was overcome by breeding tomato varieties carrying combinations of *Tm-1* with *Tm-2* or *Tm-2^2^* [21]. This decreased the likelihood of emergence of viable resistance-breaking viral mutants since multiple mutations would need to occur in the viral genome, with a consequent fitness cost, and this combined resistance went largely unbroken for many years [21]. Section 2 describes how the tension between decreased viral fitness and the domination of commercial production by resistant varieties influenced the emergence of tomato brown rugose fruit virus (ToBRFV).

**Figure 2:**
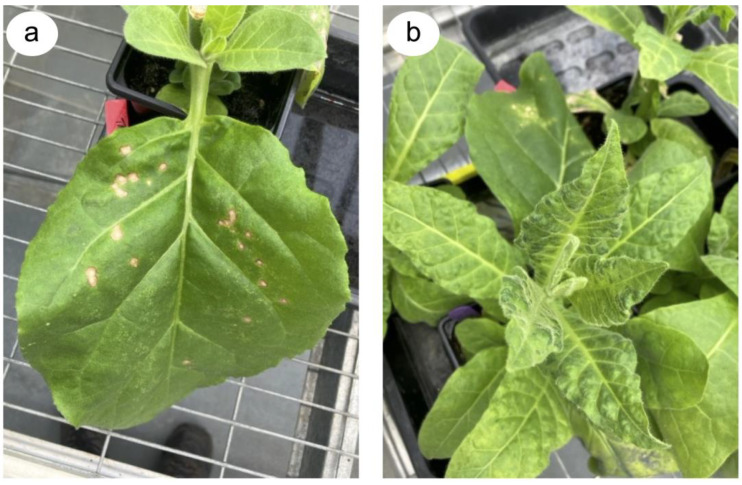
Responses of tobacco plants to a tobamovirus. Plants of tobacco (*Nicotiana tabacum*) cultivars Xanthi-nc (**a**) and Petit Havana SR1 (**b**) were inoculated on lower leaves with tomato mosaic virus (ToMV). Panel (**a**) shows a directly inoculated leaf 6 days following inoculation with ToMV. The tobacco cultivar Xanthi-nc possesses the *N* resistance gene and necrotic lesions, characteristic of the hypersensitive resistance response, have appeared at the initial infection. ToMV is unable to spread beyond these lesions. Plants of Petit Havana SR1 (**b**) do not harbor the *N* gene and the virus moves systemically, causing mosaic and leaf deformation symptoms in the uninoculated leaves.

Both *Tm-2* and *Tm-2^2^* confer hypersensitive reaction (HR)-type resistance against TMV and ToMV, in which the virus is restricted to a few hundred cells around the initial point of entry [21]. HR-type resistance is sometimes made visible by the programmed cell death of most, but not necessarily all, of the infected cells, leading to necrotic lesion formation [21] (Figure 2). The *Tm-2* and *Tm-2^2^* genes both encode immune sensor proteins of the NB-NRR (Nucleotide-Binding and Leucine-Rich Repeat domain) type. NB-LRR proteins facilitate the formation of immune signaling complexes upon directly or indirectly detecting a pathogen-derived factor, which is sometimes referred to as an ‘elicitor’ [22,23]. Elicitor recognition triggers the localized cell death response and stimulates several defensive signal transduction pathways leading to restriction of further viral spread [23,24]. For both the Tm-2 and Tm-2^2^ NB-LRR proteins, the resistance elicitor is the viral movement protein (Figure 1). Deployment of the *Tm-2* and *Tm-2^2^* resistance genes did much to gradually improve the protection of commercial tomato cultivation against tobamoviruses, and the protection they provided went largely unbroken for around 60 years until the advent in 2014 of ToBRFV. Nevertheless, in low-to-medium income countries, where older tomato varieties are sometimes cultivated, TMV and ToMV remain serious agricultural problems in smallholder farming systems (A.M. Murphy, G. Bwire, M. Ssemakala and P. Wasswa, Personal Communication).

In tobacco, the most notable tobamovirus resistance genes are the dominant *N* and *N’* genes, which were first identified in, respectively, *N. glutinosa* and *N. sylvestris* (one of the progenitors of *N. tabacum*), and subsequently introgressed into tobacco by crossing [25]. Both genes encode NB-LRR immune sensors that condition HR-type resistance against tobamoviruses (Figure 2). The N protein facilitates the detection of an elicitor sequence within the replication proteins (see Section 2), whereas in plants expressing the N’ protein, the elicitation of resistance occurs in response to specific amino acids of the coat protein (CP). The *N* gene provides wide spectrum resistance against most tobamoviruses; however, the *N’* gene provides protection against only certain tobamoviruses. For example, the *N’* gene protects against ToMV but not against the common (U1) strain of TMV [25,26,27,28]. Although it was in tobacco cultivation that TMV was originally identified as a problem, *N*-gene-mediated resistance to tobamoviruses has not been introduced universally into commercial varieties, which means that tobacco products are important sources of TMV inoculum for infection of other crops [29] (see Section 2).

Tobamoviruses are still among the major viruses affecting the production of pepper and can occur in single or mixed infections with members of other virus groups [30]. However, many pepper varieties have been protected against tobamoviruses by introduction of various *L* gene alleles which condition HR-type resistance to tobamoviruses [5]. These *L* resistance genes encode NB-LRR immune sensors for which the elicitors are amino acid sequences within the CP orthologs produced by pepper-infecting tobamoviruses [31,32,33]. Pepper lines lacking *L* genes can be susceptible to systemic infection by a broad range of tobamoviruses that include TMV, ToMV, tobacco mild green mosaic virus, paprika mild mottle virus, and PMMoV [5,33,34]. The leucine-rich repeat domains of the L proteins encoded by different *L* alleles differ in recognition specificity, creating a hierarchy of different tobamovirus pathotypes, which can be classified depending upon whether they are controlled by or overcome the major alleles *L^1^* through *L^4^*. Thus, TMV is controllable by *L^1^* but some variants of PMMoV, the most problematic of the *Capsicum*-infecting tobamoviruses [33,34], can break resistance conferred by *L^4^*, the allele that confers resistance against the widest spectrum of tobamoviruses [32,33,35,36]. Together with the deployment of resistance genes, other much more basic measures such as improved phytosanitary measures to prevent mechanical transmission from workers’ hands, tools and other equipment, as well as cultivation under protected conditions, i.e., under glass or in tunnel houses, have also helped by excluding inoculum from susceptible crops. Thus, by the early twenty-first century, the tobamoviruses had, for the most part, become a less significant threat to crop production, especially for tomato and pepper.

## 2. Why We Need Rapidly Deployable Approaches to Counter the Emergence of Resistance-Breaking and Other Emerging Tobamovirus Threats

The current resurgence of interest in the protection of crops against tobamoviruses was ignited by the reports in 2014 and 2015 in Israel and Jordan of a novel tobamovirus, ToBRFV, that could overcome the *Tm-2^2^* and *Tm-2* resistance genes, and that was not inhibited by the *Tm-1* or *tm-1* gene products [37,38,39,40]. The host range of ToBRFV is broad and includes, among other solanaceous crops, pepper and tobacco, as well as a range of non-solanaceous hosts [37,41,42]. However, it is in tomato that ToBRFV has so far caused the most significant losses in productivity (c. 70%) and in quality, by eliciting symptoms including, inter alia, leaf deformation, fruit discoloration, wrinkling (rugosity) and uneven ripening [43,44].

Phylogenetically, ToBRFV is more closely related to TMV than to ToMV [37], but with respect to the ability of ToBRFV to break resistance, the most critical differences between ToBRFV and these other tobamoviruses lie in the sequences of their respective movement proteins (Figure 1). The ToBRFV movement protein gene sequence contains mutations affecting the C-terminal protein sequence and experiments in susceptible and *Tm-2^2^* genotype tomato showed that this movement protein variant enables ToBRFV to evade resistance. But it is notable that the ToBRFV movement protein is less efficient in facilitating virus intercellular movement in susceptible plants than either of the TMV or ToMV orthologs [45,46]. Despite this loss of fitness in susceptible tomato plants, the ability of ToBRFV to overcome resistance has enabled it to out-compete TMV and ToMV because host populations have become dominated by plants carrying *Tm-2^2^* (Section 1). This competitiveness in plant populations protected against TMV and ToMV, combined with its high infectivity, has helped ToBRFV spread via the world’s trading networks to almost all tomato-growing regions, with the present exception, at least currently, of Australia [47,48].

Tobacco plants carrying the resistance genes *N* and *N*′ are resistant to ToBRFV [49], but pepper varieties carrying the *L* resistance gene alleles *L^1^*, *L^3^* and *L^4^* are transiently susceptible to systemic infection by ToBRFV, meaning that the *L* resistance gene system has limited utility in protecting pepper against this virus [36,50]. Pepper varieties lacking *L* gene alleles support systemic infection with ToBRFV but generally show tolerance, i.e., infected plants exhibit mild symptoms or no symptoms at all [36,50]. This means that although ToBRFV is not a significant threat to pepper cultivation, pepper varieties including those only transiently susceptible to ToBRFV may provide a reservoir of inoculum that could spread the virus to neighboring tomato crops. Additionally, the importation of infected but asymptomatic pepper plants to erstwhile ToBRFV-free areas may expand the epidemic in tomato [47]. Thus, it would be beneficial to develop ToBRFV resistance even in crops such as pepper that do not exhibit severe disease symptoms or which experience no significant losses in response to the virus. Interestingly, it was a similar logic that led F.O. Holmes to investigate the breeding of TMV resistance in tobacco; that is, the investigation of the *N* gene was not primarily directed to the protection of tobacco crops, but rather to decrease the inoculum reservoir in tobacco products to diminish the likelihood of TMV transmission to pepper and tomato crops (as related in ref. [29]).

The rapid spread of ToBRFV from the eastern Mediterranean region to the Americas, Europe, and Asia within the short period since 2014 is striking and has led Salem and colleagues [42] to unambiguously describe it as a ‘pandemic’. However, ToBRFV is not the only tobamovirus on a trajectory towards pandemic-level distribution. The inexorable worldwide spread of cucumber green mottle mosaic virus (CGMMV) has been slower than that of ToBRFV but is concerning for cucurbit producers. Although CGMMV has a relatively narrow host range, with most of its hosts being cucurbits, these hosts nevertheless include major cash crop plants such as cucumber, melon and watermelon, pumpkin and squash [51].

The first observations of CGMMV, or at least of the symptoms it engenders in cucumber (*Cucumis sativus*) (‘green-mottle mosaic disease of cucumber’), were reported in south and western England in the early 1920s with the first formal identification of two CGMMV strains (‘cucumber viruses 3 and 4’) as causative agents in 1935 [51,52]. Over the subsequent fifty years to 1985, CGMMV spread relatively slowly through western Europe but was eventually reported in Japan, India and the Middle East. However, the pace of spread increased through the remainder of the 20th century and into the start of the present so that by 2006 CGMMV was present in southern Europe, China and southeast Asia. CGMMV has since become established in Australia and north America, including Mexico [51,53]. At the time of writing, there do not appear to be reports of the virus in south America, but intriguingly there is a report of suspected CGMMV in samples of the grass *Deschampsia antarctica* collected on islands neighboring the Vernadsky Antarctic research station [54].

Although several weed species may serve as infection reservoirs for CGMMV, seed transmission is considered to be a major means of dissemination since the virus retains infectivity in dehydrated cucurbit seed [51]. The seedborne transmission of CGMMV provides a difficult challenge for seed producers, traders, and exporters [55] and has likely provided a major route for the virus into new areas such as the US and Asia [56,57,58]. Once established in a new region, the main local dissemination mechanism for all tobamoviruses is contact transmission, which, in commercial cultivation, occurs through inadvertent transfer of inoculum by workers, or use of contaminated tools and machinery, which is very hard to combat except through the most rigorous phytosanitary procedures [43,44].

Unfortunately, another challenge to the control of CGMMV is that it may be transmissible between melon and cucumber plants by honeybees (*Apis mellifera*), which is an unusual mode of plant virus transmission [59]. The reason this is a potentially very serious problem is that cucurbits do not produce fruit without floral visitation by a range of bee pollinators, which includes not only honeybees but also various bumblebee and solitary bee species [60]. This utter dependence on pollinators for fruit production would make it difficult to control this mode of CGMMV transmission. An additional complication is that certain viruses have been shown to influence bumblebee (*Bombus terrestris*) visitation to, and pollination of, infected hosts in ways that affect seed production and pollen transfer [61,62,63]. For bee-transmitted viruses, such effects might serve to promote the transfer of inoculum via contamination of the pollinator body or increased pollen-borne transmission (discussed in ref. [64]). At this time, however, it is unknown if CGMMV or any other tobamovirus can exert effects on host–pollinator interactions. This is an important knowledge gap that is worth investigating since virus-induced manipulation of host–bee interactions could potentially influence CGMMV epidemiology.

Tomato is not as utterly dependent on pollinator visitation for seed and fruit production as are cucurbits. However, maximal tomato fruit production requires ‘buzz’ pollination of the flowers, which can be provided only by larger bees such as bumblebees that are capable of releasing pollen through vibration, or through artificial pollination using mechanical vibration ‘wands’ [65,66]. To ensure that tomato crops attain the best possible yield and quality, plants are provided with commercially produced nests of bumblebee pollinators, which are especially important for crops grown under cover. The older approach, in which flowers must all be manually vibrated with wands, is time- and labor-intensive, and consequently far more expensive [66]. An alarming report showed that bumblebees can become contaminated with infectious ToBRFV after contact with infected tomato plants [67]. Fortunately, despite their ability to acquire inoculum on their bodies, bumblebees do not act as vectors and, although ToBRFV can infect tomato pollen, the germination efficiency of infected pollen grains is inhibited by a third [68]. If bumblebees had proven to be efficient ToBRFV vectors, this would have curtailed the widespread use of bumblebees as pollinators in commercial tomato cultivation and forced the industry to re-adopt expensive mechanical methods to maintain yields.

In this section, we have seen that tobamoviruses pose new threats to crop production that have either overcome hitherto effective genetic resistance (ToBRFV), or for which resistance is limited (CGMMV). It is timely, therefore, to explore and in some cases re-explore some very effective biotechnological options already available for the control of tobamoviruses and determine what other, more novel approaches can be deployed in the near term to aid against the re-emergence of tobamoviruses as major threats to crop production.

In the following sections, the focus will be on measures that are directly relevant to the tobamoviruses and not on broader forms of resistance, such as chemicals that trigger systemic acquired resistance or that prime induced systemic resistance. Although these may inhibit infection by tobamoviruses, they do so with less specificity, as well as with potential growth or fitness costs for treated plants. These approaches are dealt with in several recent review articles, to which readers are referred [23,69,70].

## 3. A Potential Role for Cross-Protection in Protecting Crops against Emerging Tobamoviruses

Cross-protection of tomato crops against TMV and ToMV once provided an effective control measure before the wider deployment of resistance genes (Section 1). In principle, the approach of pre-infecting seedlings with mild tobamovirus strains should be able to provide at least an interim means of protecting against PMMoV, CGMMV, or ToBRFV whilst resistant plant lines are developed. An attenuated CGMMV mutant created in planta by a combination of ultraviolet irradiation, chemical treatment and temperature stress was used in Japan for cross-protection of melon against CGMMV [71]. One of the nine substitution mutations occurring in this CGMMV strain (CGMMV-SH33b) affected the RNA silencing suppressor activity of its 126/183 kDa ortholog and it was concluded that protection against secondary infection by more virulent CGMMV strains was mediated by the induction of RNA silencing against conserved viral RNA sequences [72]. Although it is possible to sequence viral genomes produced by random mutagenesis, it may be safer and faster to design a protective strain from first principles. That is, a protective strain produced by site-specific mutagenesis of a viral infectious clone should have properties that are predictable and easier to analyze prior to release [73]. An early example of the approach was used with PMMoV by introducing site-specific mutations that both attenuated systemic disease symptoms and decreased the titer of the protective strain [74]. However, inserting a single specific mutation in a viral coding sequence may put selective pressure on the protective strain, resulting in the acquisition of a compensatory mutation elsewhere in its genome. For example, a mild protective variant of TMV was produced by replacing arginine with alanine at residue 88 in the 126 kDa protein sequence, but a spontaneous mutant that caused severe symptoms (with replacement of serine with lysine at residue 114 of the 126 kDa protein) arose independently in 3 out of 14 plants inoculated with the protective strain [75]. Thus, engineered protective strains need to be investigated extensively and may need further mutations to be introduced to make reversion to a virulent form less likely.

Cross-protection with attenuated strains of ToBRFV has an inherent risk since these strains will, like wild-type ToBRFV, be able to overcome existing genetic resistance in tomato and to spread in pepper crops. Thus, a protective ToBRFV strain may have the potential to disseminate at least as widely as the wild type and, if further mutations occurred, this could result in the emergence of a novel virulent strain. There is evidence that this scenario can occur. Recently, it was reported that a suspected unauthorized attempt at cross-protection with a ToBRFV mutant may have caused the emergence of a new ToBRFV lineage, which has spread extensively in the Netherlands [76]. Elements of unpredictability with the deployment of cross-protecting strains include not only further mutation, producing a novel virulent variant, or reversion to the wild-type sequence (albeit this is less likely if more than one region of the sequence has been modified in the protective strain), but also the unintended consequence of causing disease in other crops [77], or the possibility to synergize other viruses in mixed infections with increased disease severity. This is the case for mixed ToBRFV and PepMV infections [78]. A virus can also inhibit resistance to infection by other pathogens, which has been shown with mixed infections of ToBRFV and the necrotrophic fungal pathogen *Botrytis cinerea* [79].

Despite improvements in inoculation techniques [80], infecting plants with protective strains is time- and labor-intensive. Additionally, within certain jurisdictions such as the European Union (EU), regulatory frameworks for approvals of cross-protection agents have become tighter since the heyday of cross-protection in the 1970s, and for a quarantine agent such as ToBRFV, the release of even a mild variant is illegal [76]. Therefore, with respect to the control of the current cohort of tobamoviruses threatening crops, cross-protection is at best a stopgap, and for ToBRFV, the deployment of cross-protection may have risks that outweigh any potential benefits.

## 4. Pathogen-Derived Resistance against Tobamoviruses

In one of its earliest formulations, the concept of pathogen-derived resistance to viruses arose from the idea that the expression of wild-type or mutant viral gene products in a host plant would interfere with the normal progression of the viral infection cycle without affecting the biology of the host [81]. Thus, almost any point in the cycle (virion uncoating, viral replication, viral short and long-distance movement in the host, virion assembly) was a potential target for disruption through transgenic expression of the relevant viral protein or a mutated version thereof [82]. The first successful demonstrations of the pathogen-derived resistance concept, as well as the first successful use of genetic engineering to produce pathogen-resistant plants, came from the work of R.N. Beachy and colleagues in the 1980s. These researchers showed that constitutive expression of the TMV CP in transgenic tobacco and tomato plants interfered with viral uncoating, systemic movement of the virus, and effects on the viral cell-to-cell movement protein, and they proposed that it worked via mechanism(s) similar to cross-protection (interference between related virus strains) [83,84]. The initial step of TMV infection in directly inoculated plant cells is the co-translational uncoating of the rod-shaped virus particles by host ribosomes as they simultaneously remove CP subunits and translate the 5′-proximal 126/183 kDa ORF (see Figure 1) [85]. In the *CP*-transgenic plants, CP accumulation was sufficient to somehow resist this process and delay the establishment of infection when virus particles were used as inoculum, but when naked TMV RNA was used, the transgenically expressed CP was not sufficiently abundant to encapsidate the RNA and prevent its translation [86,87]. The approach of expressing viral CPs (and subsequently other viral gene products) [88,89] was soon extended to other viruses. Remarkably, in transgenic plants expressing the CPs of TMV or alfalfa mosaic virus (AlMV), there was some protection against unrelated viruses. The protection was manifested as a short delay in disease development, presumably caused by *trans*-encapsidation of the RNA of the unrelated viruses by the TMV or AlMV CP [90]. Early experiments in crop plants included demonstration of CP-mediated resistance to CGMMV in transgenic watermelon [91] and against various viruses in potato, tomato and cucurbit crops [92,93,94]. The expression of another tobamovirus gene product, the 54 kDa region of the replicase protein (Figure 1) in transgenic tobacco plants, provided strong immunity against TMV [95]. This ‘replicase-mediated’ resistance approach also worked in crop plants, as shown in the protection of transgenic cucumber against the tobamovirus cucumber fruit mottle mosaic virus by the expression of the orthologous 54 kDa protein [96]. This period of research provided the first demonstrations that genetic engineering can protect crops very effectively against viruses.

The original *CP*-transgenic tobacco lines created by Beachy and colleagues were resistant to TMV by virtue of CP-mediated inhibition of both uncoating and systemic movement. These plant lines provided a clear demonstration of the feasibility of the initial concept of pathogen-derived resistance, i.e., a resistance mechanism that works due to interactions of viral or virus-derived components that are independent of any pre-existing host defenses. Indeed, these plants exhibited no apparent changes in the accumulation of pathogenesis-related proteins, which were among the very few resistance induction markers available in the late 1980s [97]. However, as work on pathogen-derived resistance advanced and was extended to other viruses and to other viral gene products (replicase proteins, movement proteins, etc.) [23,88,89], it became apparent that resistance in some transgenic plant lines was due to the triggering of an entirely unsuspected natural antiviral mechanism in which infection induced the production of small RNAs that were complementary to viral sequences and that directed the cleavage of viral RNAs [98,99]. This defense system was later called (variously) RNA silencing, RNA interference or post-transcriptional gene silencing. Fortuitously, RNA silencing was later found to occur not only in plants but in most eukaryotic organisms, and with many important functions in addition to antiviral defense. Its study has led to many key discoveries with practical biotechnological and biomedical applications [10,98,99].

The early studies of pathogen-derived resistance showed that it is not easy to predict whether expressing a virus-derived protein will engender resistance to a virus through specific protein-mediated interference with the viral infection cycle, from elicitation of antiviral silencing by the RNA transcript of the transgene, or through a combination of both effects. Given the strong and specific resistance that RNA silencing can provide, it now seems better to start with virus-derived constructs designed explicitly to prompt RNA silencing against a virus (Section 5).

## 5. Using RNA Silencing to Engender Resistance against Tobamoviruses in Transgenic and in Non-Engineered Plants

Improved understanding of the mechanism of RNA silencing in plants has allowed transgene construct design to increase in sophistication. Early examples of RNA-mediated protection against viruses were discovered by chance, for example, in seminal work on expression of translatable and non-translatable *CP* constructs derived from the potyvirus tobacco etch virus [100]. Later iterations of RNA-mediated protection were used successfully to protect papaya crops against the potyvirus papaya ringspot virus [101,102]. Designing transgenes to constitutively express double-stranded virus-derived RNA sequences, for example, by expressing transcripts that fold into hairpin structures (i.e., containing complementary plus-sense and minus-sense virus-derived RNA sequences separated by a short spacer region), renders them liable to cleavage into double-stranded short-interfering RNAs (siRNAs) by the same Dicer-like (DCL) endonucleases that participate in naturally occurring antiviral RNA silencing [99]. Once these transgene-derived RNA transcripts have been rendered into a single-stranded form, they base-pair to complementary viral RNA sequences and facilitate sequence-specific cleavage by Argonaute (AGO) endoribonucleases. Such approaches have been shown to be effective in inducing resistance against TMV and PMMoV in stably transformed plants and in transient expression systems [103,104,105,106].

Another class of small RNAs directing AGO-mediated sequence-specific RNA cleavage are microRNAs (miRNAs). Unlike siRNAs, which are generated de novo, plant miRNAs are encoded by nuclear genes and generated from precursor transcripts by DCL-mediated cleavage. The predominant role of plant miRNAs is in preventing excess accumulation of specific cellular mRNAs, particularly those encoding highly potent factors regulating defense, stress responses and development; such proteins, which include transcriptional regulators, could be potentially harmful if their levels were not subject to tight regulation [107,108,109]. Artificial miRNAs (amiRNAs) are generated by replacing part of the sequence of naturally occurring miRNAs such as miR171 or miR159a/b. For example, a short sequence that is complementary to a section of viral RNA can be inserted into a miRNA backbone to re-direct AGO endonucleases to attack viral RNAs. Such approaches have been reported in model plants for successful protection against turnip yellow mosaic virus, turnip mosaic virus and CMV [110]. Unfortunately, there do not appear to be published reports for production of transgenic plants expressing amiRNA-mediated protection against tobamoviruses; so, it is difficult to compare the efficacy of this approach to other RNA-mediated resistance strategies. However, an intriguing study found that some naturally occurring tomato miRNAs have potential target sequences within the ToBRFV genome [111], although it is currently unknown if these miRNAs are defensive in function.

Strategies based on the expression of RNAi-inducing molecules have proven to be highly successful in protecting plants against viruses; however, it may not always be necessary to obtain this protection through constitutive expression in transgenic plants. Several research groups have explored the triggering of antiviral RNA silencing using topical application, spraying or injection of plants with synthetic double-stranded or hairpin RNAs produced in vitro, for example, RNAs complementary to the *54 kDa* coding region of PMMoV [112] or the *126 kDa* and *CP* sequences of TMV [113]. Other methods of producing synthetic RNAs include purification from bacteria transformed with modified plasmids, for example, targeting the *MP* and *CP* coding sequences of CGMMV [114], or bacteria infected with a modified bacteriophage (*ϕ6*-infected *Pseudomonas syringae*) to target various regions of the TMV genome [115]. Using topical application of inducers of antiviral silencing is a potentially very ‘nimble’ approach for protecting crops against emerging or invasive viruses such as ToBRFV or CGGMV, since synthetic double-stranded RNA molecules can be manufactured rapidly. Importantly, in crop plants the approach would not require the transformation and selection of suitable resistant plant lines, which could be time consuming (albeit far less so than conventional breeding); instead, existing unmodified plant varieties could be retained. An additional advantage of the technology is that it can be employed in crop plant species or cultivars that are intractable to genetic transformation and for which conventional genetic engineering is therefore unfeasible.

However, a drawback of topical RNA application is the sensitivity of naked double-stranded RNA to nucleases and UV radiation, which these molecules may encounter on the surface of a plant. Various compounds have been used or proposed as nanoparticle carriers to increase the stability, penetrance and silencing induction properties of double-stranded or hairpin RNA cargoes (reviewed in ref. [116]). Carrier materials themselves may have biological effects including resistance induction. This may be useful in practice but may confound the analysis of modes of action. For example, nanoparticles derived from the fungal cell wall product chitosan were used to encapsulate bacterially synthesized dsRNA molecules derived from the *CP* sequence and a sequence within the *126/183 kDa* gene. Application of these formulations limited seedborne transmission of TMV in *N. benthamiana* and tobacco [117]. Although true seedborne transmission of TMV only occurs at a low frequency [118], the results are interesting. However, it should be noted that chitosan is a potent inducer of resistance mechanisms other than RNA silencing [69,119], which may have contributed to the success of the approach. More recently, various synthetic organic polymers with cationic properties have been investigated as protectants and slow-release agents for biologically active double-stranded RNA under challenging conditions (e.g., the plant phylloplane, the rhizosphere, insect guts, etc.) [120,121]. However, the use of these formulations has been proposed mainly for control of invertebrate pests and at this time there do not seem to be any reports of these polymers being used as carriers of antiviral double-stranded RNA molecules.

Probably the most successful carrier used so far appears to be layered double hydroxide (LDH) anionic clay nanoparticles [122]. Synthetic double-stranded RNA to target the *54 kDa* sequence (based on that developed previously by Tenllado and Diaz-Ruiz [112]) was incorporated into LDH nanoparticles and provided protection against PMMoV, which was monitored by measuring HR lesion numbers on *NN*-genotype tobacco leaves. Protection lasted for 20 days following application for double-stranded RNA incorporated into LDH nanoparticles, compared to only 5 days for naked double-stranded RNA [122]. Work with LDH nanoparticles loaded with double-stranded RNA directed against CMV indicated that the nucleic acid cargo can penetrate plant tissues and that treatment can stimulate resistance systemically, and even in newly emerging leaves [122]. Subsequent work demonstrated the versatility of the LDH nanoparticle system by showing that it can be used to inhibit insect-mediated virus transmission, as well as control the insect vectors themselves [123,124], which demonstrates the versatility of the technology. Given the initial positive results with PMMoV [122], further work with other tobamoviruses and LDH nanoparticles should be carried out since it may have significant potential for developing an effective means of ameliorating the impact of emerging tobamovirus threats. An advantage of the approach is that LDH formulations containing mixtures of double-stranded RNAs targeting multiple tobamovirus sequences could be used to control several tobamoviruses simultaneously and might help inhibit the emergence of resistance-breaking viral mutants.

Much remains to be understood regarding the modes of action and any additional effects for materials that have been developed or proposed as carriers for double-stranded RNA and application methods still need to be perfected in some cases. It is still not clear in detail how topically applied RNA molecules enter plant tissues to trigger silencing, and some approaches to spray application may not be compatible with the approach due, for example, to damaging of the double-stranded RNA cargoes [125]. Further refinements in carrier materials and procedures for application will likely overcome these problems [116].

## 6. Potential for Interspecies Transfer of *R* Genes, Rescue of ‘Broken’ Resistance and Creation of Novel Resistance Factors

As described in Section 1 and Section 2, in several host species, resistance genes have evolved to protect them against tobamoviruses. Possibly the most broadly effective of these is the *N* gene that was introgressed, with some difficulty [29], from *N. glutinosa* into several lineages of *N. tabacum* [25]. With the documented exception of the Hungarian Ob strain of ToMV (Solanum dulcamara yellow fleck virus Ob) [126,127], the *N* gene provides strong HR-type resistance against almost all tobamoviruses. The *N* gene product is a TOLL/INTERLEUKIN-1 RECEPTOR-LIKE domain immune sensor protein that mediates the recognition of a protein sequence that comprises a region of amino acid residues (c. 692–1116) of the 126/183 kDa ORF that encompasses the RNA helicase/ATPase domain (Figure 1) [128]. The current knowledge of the mechanism by which the N protein mediates host responses following detection of this viral protein sequence will not be dealt with here since it has been described in detail in a recent review by Palukaitis and Yoon [23].

Soon after they had isolated the *N* gene, Baker and colleagues showed that it could be transferred to another species, tomato [129,130]. In tomato, the introduced *N* transgene conferred resistance against TMV with the appearance of HR lesions at infection sites and with no spread of the virus to uninoculated parts of the plant [130]. The *N* gene was also transformed into *N. benthamiana,* where it conditioned HR-type resistance against wild-type TMV and limited the spread of a modified TMV expressing the green fluorescent protein [131]. Work of this kind showed that introducing the *N* gene, or potentially other dominant *R* genes (such as *Tm-2* or *Tm-2^2^* or the *L* genes), to heterologous backgrounds could provide practical and effective protection against tobamoviruses. Furthermore, the introduction of these *R* genes by genetic engineering is precise and diminishes the potential for yield penalty. For example, in some commercial ‘flue-cured’ *NN* genotype tobacco lines that were produced through conventional crossing, yield penalties were reported and have been attributed to co-introgression of deleterious *N. glutinosa* DNA sequences closely linked to the *N* locus [29,132]. The successful transfers of the *N* gene to transgenic tomato and *N. benthamiana* plants and the demonstration that the transgene conferred full resistance against wild-type TMV implied that all the molecular partners required for the N protein to function are conserved, at least between solanaceous plants. The introduction of the *N* gene into transgenic versions of commercial tomato would have a major impact against the emerging threat of ToBRFV since we know that the transfer of the *N* gene into this crop is feasible and that ToBRFV cannot overcome this resistance gene [49].

Due to the limited host range of CGMMV, it has not been possible to determine directly if either the *N* or *L* resistance genes will provide any protection against the virus. However, given the effectiveness of the *N* gene and of the *Capsicum*-origin *L* resistance genes in providing protection against so many tobamoviruses, inter-species transfer and further modification (see below) could conceivably be used to protect vulnerable cucurbit crops against CGMMV. Although most CGMMV strains do not infect plants of either *Capsicum* or *Nicotiana* (including *N. glutinosa*, the source of the *N* gene), one CGMMV isolate has been found to induce chlorotic, non-necrotic, concentrically ringed lesions on leaves of tobacco plants regardless of the presence of the *N* gene. CGMMV infection remained localized to the inoculated leaves, suggesting that the appearance of chlorotic lesions is a resistance response [51,133]. This resistance to CGMMV (which is probably stronger and symptomless for other CGMMV strains) may help explain why the host range of CGMMV does not include tobacco. Characterization of the tobacco gene(s) controlling resistance to CGMMV could enable their isolation and transfer to cucurbit host backgrounds to engender resistance to CGMMV.

It should be possible to modify *R* genes and their products or modify their interaction partners to create new resistance systems or to ‘rescue’ existing *R* gene systems that have been overcome by the emergence of resistance-breaking tobamoviruses. For example, it was shown that random mutagenesis products for an *R* gene encoding a NB-LRR, specifically the *Rx* gene for recognition of the potexvirus potato virus X (PVX), could be selected to obtain variants conferring resistance to a wider range of PVX strains, and even resistance against a more distantly related potexvirus, poplar mosaic virus (PopMV). Further mutation and selection of sequences encoding PopMV-specific Rx protein variants allowed for optimization of the resistance to prevent trailing necrosis associated with a ‘leaky’ HR-type defense reaction [134,135]. One can envisage using this approach to screen a library of randomly mutagenized *R* gene variants to identify those capable of conferring resistance viral isolates able to break resistance conferred by the parental *R* gene. Hypothetically, clones of the current range of *R* genes used to protect tomato against tobamoviruses, i.e., *Tm-1* (and its allelic factor *tm-1*), *Tm-2* and *Tm-2^2^*, could be subjected to mutagenesis and screened in *N. benthamiana* transient assays for restoration of resistance to ToBRFV. Characterization of the key amino sequence changes that have restored resistance will enable the use of gene editing in tomato to selectively introduce the appropriate changes in the sequences of the pre-existing *R* genes. Gene editing with the Crispr-Cas 9 (clustered regularly interspaced short palindromic repeats/CRISPR associated protein 9) system is feasible in tomato [136,137] (and in cucurbits) [138], making this a technically viable approach to renewing resistance to tobamoviruses in existing cultivars.

Gene editing was successfully used to mutate members of a gene family encoding a key host factor needed to support tobamovirus replication, TOM1 (TOBAMOVIRUS MULTIPLICATION 1) [136]. TOM1 is a seven-pass transmembrane protein that anchors tobamovirus replicase complexes to the tonoplast and other intracellular membranes and, together with an associated small GTP-binding protein (ARL8), is essential for successful replication [139,140,141]. TOM1 was originally identified from a screen of *Arabidopsis thaliana* for mutants (*tom* mutants) with decreased susceptibility to ToMV and a crucifer strain of TMV (TMV-Cg, also known as youcai mosaic virus, YoMV) [142]. Tomato TOM1 protein orthologs are encoded by a small four-member gene family and are also susceptibility factors for tobamovirus infection. It was found that using Crispr-Cas 9 to mutate all four gene family members had no apparent deleterious effects on plants but rendered them immune to TMV, ToMV and YoMV, while having no effect on susceptibility to infection by PVX or the Cucumovirus tomato aspermy virus [136]. *TOM1* orthologs are conserved across a very broad taxonomic range of plants [143]. In addition to TOM1, mutant screens have identified other factors supporting tobamovirus replication including TOM3 (another transmembrane protein that supports tobamovirus replication) and TOM2A (which plays a subsidiary role in supporting tobamovirus replication). Knock-out of *TOM1* (paralog *TOM1a*) and *TOM3* by gene editing in an otherwise tobamovirus-susceptible tomato background rendered plants resistant to ToBRFV, although knockouts of the genes singly were less effective [137]. Overall, editing of *TOM1* and *3* orthologs, especially if carried out for all paralogs, could provide a means of protecting not only tomato but also other crops, such as pepper and cucurbits.

In addition to the TOM factors, there are a variety of host factors that may play roles in supporting tobamovirus infection but are far less well understood [144]. These other host factors are deserving of further study as they could provide targets for gene editing to produce plants able to resist (or at least inhibit) infection. Such factors influence tobamovirus gene expression (e.g., the translation factors eEF1A or the eIF3-associated factor GCD10, which in yeast is associated with initiator tRNA stabilization) [145,146,147]; the localization of viral proteins within infected cells (e.g., the auxin/indole-3-acetic acid family of proteins) [148]; intercellular movement via the plasmodesmata, e.g., plasmodesmal pectin methylesterase or the membrane trafficking protein synaptotagmin A, which both interact with tobamoviral movement proteins [149,150,151]; or factors that control systemic movement through the phloem such as *vsm1*, glycine-rich proteins, and callose-binding proteins in phloem cell walls [152,153,154].

Testing novel candidate genes for protection against tobamovirus infection requires a good testbed, which is *N. benthamiana*. As alluded to in the above discussion of *R* gene variants, agroinfiltration-mediated transient expression in *N. benthamiana* provides a facile method for high-throughput screening to identify and characterize potential tobamovirus resistance or susceptibility factors, as demonstrated for PMMoV [155]. Additionally, *N. benthamiana* lends itself to the identification of factors affecting tobamovirus infection since the most used lab strain (accession RA-4) appears to be susceptible to all tobamoviruses [156,157].

## 7. Why There Is a Future for New (and Old) Methods for Protection and against Tobamoviruses

We have seen from the foregoing sections that the capability to produce genetically engineered lines of tomato, pepper and cucurbits with strong resistance to tobamoviruses has existed since the 1980s and 90s. Why did the remarkable achievements of that period not revolutionize the protection of crops against viruses, and why are we now faced with the re-emergence of tobamoviruses as serious threats to the production of these high-value crops?

One might try and explain this puzzle using some well-worn arguments, for example, by criticizing anti-GM non-governmental organizations and the public and political hesitancy about genetic engineering that they helped engender. This is true to some extent. Greenpeace, although no doubt well-intentioned, has had a baleful influence in slowing the deployment of some transgenic crops, such as vitamin-A-enriched transgenic rice, which has the potential to benefit millions of malnourished people [158,159]. It is also common to blame onerous and overly burdensome regulatory frameworks for regulation of genetic engineering that are imposed in certain countries or regions. The most notable example typically cited is the EU, where a recent legal judgement threatens to inhibit the use of gene editing [160]. While these social movements and political trends may have played roles in slowing the deployment of some genetically engineered crops, they are unlikely to fully explain why genetic engineering has not been used to consign tobamoviruses (and many other plant viruses) to history. This is because since around 1996, other plants which have been genetically engineered for various traits have, with apparent inexorability, increased, such as a proportion of maize, cotton, soybean, and eggplant (also known as aubergine and brinjal) crops, and, in some regions, genetically engineered lines dominate production [161]. Many of these crop plants are engineered to express *Bacillus thuringiensis* Cry (BT) toxin and vegetative insecticidal proteins to protect against a range of lepidopteran and coleopteran pests, and in the Americas and Asia, these insect-resistant plants constitute the majority of cotton and maize production. Even in the EU, by 2019, 36 and 6% of the maize grown in Spain and Portugal, respectively, was genetically engineered for insect pest resistance [161].

I suggest that the most likely specific cause for the non-adoption of the highly successful pathogen-derived resistance strategy for generating tobamovirus-resistant transgenic plants (Section 4) and for the limited exploration of the potential for inter-species transfer or optimization of tobamovirus resistance genes (Section 5) lies in a combination of economics and unfortunate timing. With respect to tomato and pepper, for example, by the time that the commercialization of genetically engineered crops began, there were already good genetic resources available, i.e., *Tm2* and *Tm2^2^*, which provided very effective ToMV and TMV control in tomato and the *L*-genes which in various combinations provided protection against most pepper-infecting tobamoviruses. Meanwhile, in the 1980s and 90s, CGMMV had not spread to its present, virtually worldwide extent and therefore was not as important a problem for producers (Section 2). Thus, investment in using genetic engineering against tobamoviruses probably looked far less financially attractive than it would have done during the slightly earlier era of cross-protection, which is expensive to deploy at scale and which often causes some loss in yield.

With the new worldwide threats to tomato production from ToBRFV and to cucurbit production due to CGMMV, the situation has changed completely since the 1990s. Some of the biotechnological approaches described in this review could play roles in preventing serious losses from these tobamoviruses. In particular, the use of gene transfer, gene editing and the application of synthetic double-stranded RNAs to induce antiviral silencing in non-engineered plants stand out. The last of these has great promise due to its potential flexibility and speed. Whether we succeed in controlling these tobamoviruses using genetic engineering, or through some other approach, an important lesson from the emergence of ToBRFV is that a relatively ‘weak’ virus can thrive and spread very rapidly in a population of plants resistant to otherwise fitter and more competitive viruses. Thus, it is essential that in the future we do not depend upon a single strategy or on a single genetic system (i.e., two similar resistance alleles recognizing the same viral elicitor in the case of *Tm2* and *Tm2^2^*) for crop protection against tobamoviruses since this will eventually drive the emergence of new and possibly more destructive versions of these old viral enemies.

## Data Availability

No new data were created or analyzed in this study. Data sharing is not applicable to this article.
